# Research on the Effect of Calcium Alginate-Red Mud Microspheres on the Performance of Cement Mortar by Partially Replacing Standard Sand

**DOI:** 10.3390/ma18143326

**Published:** 2025-07-15

**Authors:** Ruizhuo Liu, Zibo Lin, Shencheng Fan, Yao Cheng, Yuanyang Li, Jinsheng Li, Haiying Zou, Yongsi Chen, Liting Zheng, Jing Li

**Affiliations:** 1 School of Chemistry and Chemical Engineering, Guangxi University, Nanning 530004, China; 2204110133@st.gxu.edu.cn (R.L.); 2214391062@st.gxu.edu.cn (Z.L.); 2414402237@st.gxu.edu.cn (S.F.); 2204110538@st.gxu.edu.cn (Y.C.); 2204110103@st.gxu.edu.cn (Y.L.); 2204110117@st.gxu.edu.cn (J.L.); 2204110114@st.gxu.edu.cn (H.Z.); 2204110122@st.gxu.edu.cn (Y.C.); 2 Key Laboratory of New Low-Carbon Green Chemical Technology, Education Department of Guangxi Zhuang Autonomous Region, Nanning 530004, China

**Keywords:** red mud, sodium alginate, cement mortar, artificial sand, mechanical properties

## Abstract

With the depletion of river sand resources and increasing environmental concerns, the development of alternative materials has become an urgent need in the construction industry. Waste concrete and non-waste concrete materials have been widely studied as alternatives to river sand. Although recycled concrete fine aggregates are close to natural sand in terms of mechanical properties, their surface cement adheres and affects the performance of cement, whereas non-recycled concrete fine aggregates perform superiorly in terms of ease of use and compressive properties, but there are challenges of supply stability and standardization. Red mud, as an industrial waste, is a potential alternative material due to its stable supply and high alkaline characteristics. In this paper, a new method is proposed for utilizing the cross-linking reaction between sodium alginate and calcium chloride by the calcium alginate-red mud microsphere preparation technique and the surface modification of red mud to enhance its bonding with cement. The experimental results showed that the mechanical properties of CMC-RM-SiO_2_-2.5% were improved by 13.9% compared with those of the benchmark cement mortar, and the encapsulation of red mud by calcium alginate significantly reduced the transfer of hazardous elements in red mud.

## 1. Introduction

River sand has been widely utilized in modern construction due to its favorable particle morphology. However, with the continuous population growth and accelerated urbanization, the increasing demand for river sand in the construction industry has led to its gradual depletion. Furthermore, excessive river sand extraction disrupts the natural fluvial ecosystem, resulting in environmental issues such as riverbed erosion [1]. Therefore, identifying effective substitutes for river sand, while ensuring construction quality, has become a pressing challenge.

Against the backdrop of restricted river sand mining, numerous studies have been conducted to develop suitable alternatives [2,3,4,5,6]. These alternatives are typically categorized as recycled concrete fine aggregates (RCFA) derived from waste concrete and recycled non-concrete fine aggregates (RNCFA) produced from non-concrete materials such as bricks, glass, and ceramics. RCFA offers several advantages, including stable and abundant sources, potential environmental benefits, and favorable mechanical properties. Ali Akhta [7] reported that more than 4 billion tons of waste concrete are generated annually worldwide, providing a substantial resource base for RCFA production. Hossain et al. [8] and Verian et al. [9] revealed that utilizing RCFA as construction aggregate not only reduces landfill costs but also decreases carbon emissions by 65% and energy consumption by 58%. Additional studies have demonstrated that concrete incorporating RCFA can achieve mechanical performance comparable to that of concrete made with natural river sand. Beltrán et al. [10] reported that adjusting the water-to-cement ratio and increasing the cement dosage can enhance the mechanical strength of RCFA concrete. Moreover, optimized particle grading [11] and advanced mixing techniques [12] have also been shown to significantly improve RCFA concrete properties. Despite its promising prospects, RCFA exhibits certain drawbacks, such as residual adhered cement paste on the surface, which impairs mechanical performance [13], and higher water absorption, which adversely affects workability [14]. These limitations warrant careful consideration. In this regard, the use of RNCFA can effectively circumvent these issues. J.M. Khatib et al. [15] compared recycled brick aggregate and recycled concrete aggregate in concrete applications, finding that concrete incorporating recycled brick exhibited superior long-term strength and shrinkage performance, with reduced strength degradation and significantly lower shrinkage than its RCFA counterpart. Soliman et al. [16] indicated that the low water absorption and potential pozzolanic activity of waste glass sand enhance the workability and compressive strength of concrete. Nonetheless, despite its performance advantages, RNCFA suffers from supply instability and a lack of standardization.

To overcome these challenges, researchers have turned their attention to alternative materials, among which red mud, an industrial waste by-product, has attracted increasing interest. Red mud (RM) is an insoluble solid waste generated during alumina production via the Bayer process, characterized by a high alkalinity, fine particle size, and complex mineral composition. In China alone, over 100 million tons of red mud are produced annually, ensuring a stable supply. Common techniques for converting red mud into aggregates include pan granulation with subsequent curing methods such as high-temperature sintering and alkali activation. Sun et al. [17] employed pan granulation followed by sintering to improve aggregate strength, while Zhao et al. [18] enhanced strength by spraying alkali activators onto dispersed red mud particles. However, both methods present notable drawbacks: high-temperature sintering is energy-intensive and environmentally unfriendly [19,20], whereas alkali activation demands the precise control of the activator composition and involves complex operations [21]. Hence, this study proposes a simplified approach by granulating red mud through the cross-linking reaction between sodium alginate and calcium chloride to prepare calcium alginate–red mud microspheres. To enhance the interfacial interaction between the microspheres and cement matrix, the red mud surface was modified using a silane coupling agent and grafted with nano-silica. This method not only simplifies conventional granulation processes but also facilitates red mud encapsulation, thereby mitigating potential environmental hazards. The silane-nano-silica modification further improves bonding with cementitious materials and enhances the properties of the microspheres.

## 2. Materials and Methods

### 2.1. Raw Materials

The red mud used in this study was sourced from an alumina production company in Guangxi, China. Sodium alginate and nano-silica were supplied by Aladdin Reagent Co., Ltd., Shanghai, China. All experimental materials were sieved using a 100-mesh screen. P.I 42.5 cement was produced by Fushun Cement Co., Ltd., Fushun, China and ISO standard sand was obtained from Xiamen Essiso Standard Sand Co., Ltd., Xiamen, China The chemical compositions of red mud and cement were analyzed using BRUKER S8 TIGER XRF analysis (Bruker Corporation, Karlsruhe, Germany), as shown in Table 1. 

**Figure 1:**
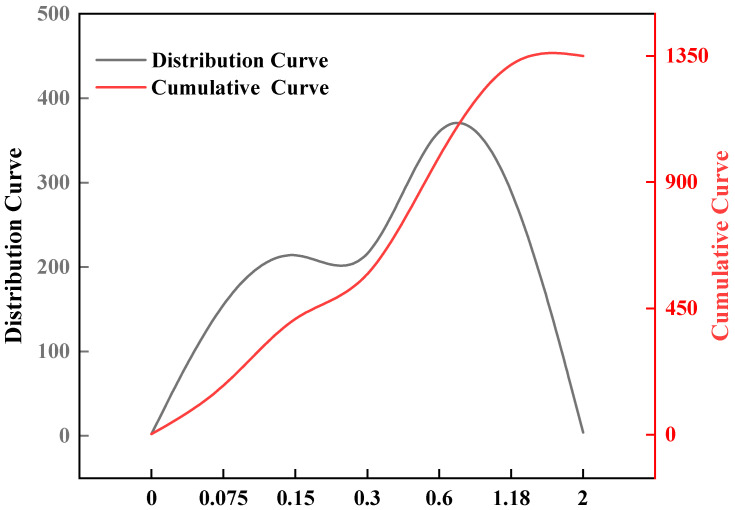
Sieving curve and cumulative curve.

The particle size distribution (by vibrating machine ZBSX-92A, Cangzhou, China) of the standard sand is presented in Figure 1, indicating that the dominant particle size ranges from 0.6 mm to 1.18 mm.

### 2.2. Preparation and Modification of Calcium Alginate–Red Mud Microspheres

**Figure 2:**
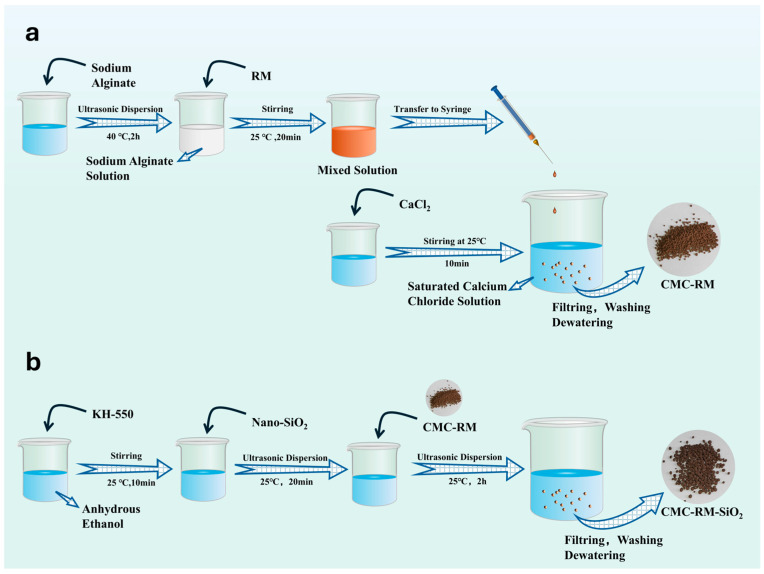
(**a**) Red mud microsphere preparation; (**b**) grafting process.

The preparation process of calcium alginate–red mud microspheres is illustrated in Figure 2.

(1) In total, 10 g of sodium alginate powder was dissolved in 500 mL of deionized water under ultrasonic agitation for 4 h to obtain a bubble-free solution. Then, 50 g of red mud was added and stirred thoroughly, followed by 2 h of ultrasonication to yield a sodium alginate–red mud suspension.
(2) The suspension was dripped into a saturated calcium chloride solution using a syringe, forming hydrogel microspheres, which were filtered and rinsed with water to remove residual CaCl_2_ and then dried in an oven at 65 °C to a constant weight.
(3) In total, 20 mL of KH-550 silane coupling agent was dispersed in 100 mL of ethanol, into which 2 g of nano-silica and 10 g of the dried microspheres were added, followed by 2 h of ultrasonication.
(4) The modified microspheres were filtered, washed with deionized water to remove unreacted KH-550 and silica, and oven-dried at 65 °C to a constant mass.

### 2.3. Preparation of Modified Cement Mortar

The calcium alginate modified cement (CMC) was prepared by partially replacing standard sand with red mud microspheres at substitution rates of 2.5%, 5%, 7.5%, and 10% by weight, as summarized in Table 2. Before mixing, the standard sand was sieved to remove particles larger than 2 mm. The appropriate quantities of red mud microspheres or modified microspheres were weighed according to Table 2 and mixed with the remaining standard sand. Control Check (CK) cement mortar was prepared by adding 450 g of cement and 225 g of water into a mixer and stirring for 4 min. During the last 2 min of mixing, the blended sand and microspheres were added. The fresh mortar was cast into 40 mm × 40 mm × 120 mm molds and cured in a standard curing room at 20 ± 1 °C and 95 ± 5% relative humidity for 1 day before demolding. Subsequently, the specimens were cured for 7 and 28 days.

### 2.4. Testing and Characterization Methods

#### 2.4.1. Mechanical Properties

Mechanical properties were evaluated using a universal testing machine CDT1305-2 (Wuxi Jianyi Instrument & Machinery Co., Ltd., Wuxi, China), in accordance with the GB/T 17671-1999 standard [22]. The flexural strength was measured at a loading rate of 60 N/s, and the compressive strength was measured at 2500 N/s. The fluidity of the cement mortar was measured using an NLD-03 cement flow table apparatus(Shanghai Guangdi Instrument and Equipment Co., Ltd., Shanghai, China), following the JTG 3420-2020 standard [23]. The flow table was operated for 25 drops per test.

#### 2.4.2. Microscopical Properties of Modified Cement Mortar

A small portion of mortar samples was crushed and dried for toxicity leaching tests using a solid-to-liquid ratio of 1:20. The hydration process was halted by ethanol immersion, followed by the oven-drying and grinding of the specimens. Functional groups were characterized via FT-IR spectroscopy. Toxicity leaching tests were conducted to assess the environmental safety of the modified mortars. Additionally, the effects of red mud microspheres on cement hydration were examined using X-ray diffraction (XRD, DX-2700A) and thermogravimetric analysis (TGA, TG209F1). The internal pore structure was analyzed by BET surface area measurements, and fracture surfaces were observed using field-emission scanning electron microscopy (FE-SEM).

## 3. Results

### 3.1. Modified Cement Mortar Characterization

#### 3.1.1. Fluidity Test of Modified Cement Mortar

**Figure 3:**
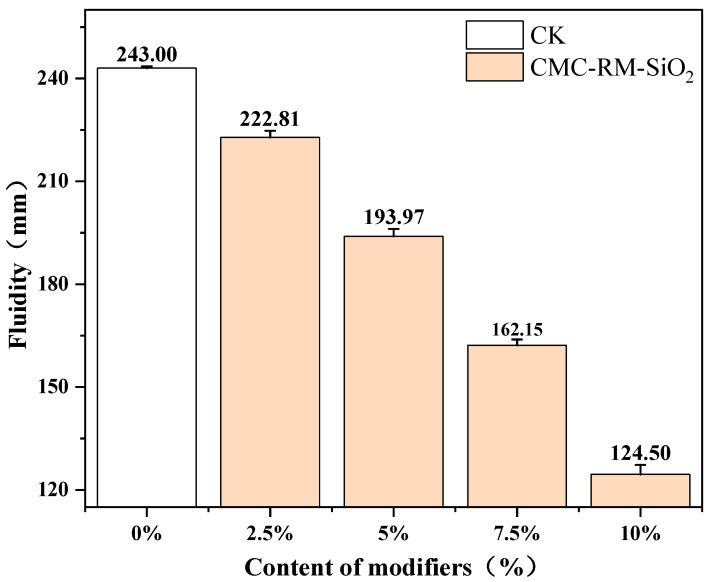
Mechanical properties of modified cement mortar.

Figure 3 presents the flowability test results for cement mortars with different sand replacement rates. It is worth noting that, as the substitution rate of red mud microspheres increased to 10%, the flow diameter of the CMC-RM-SiO_2_ mortar decreased from 243 mm to 124.5 mm, representing a 48.8% reduction. This value falls significantly below the 180 mm minimum construction requirement specified in GB/T 2419-2005 [24], thereby limiting the applicability of the modified mortar in practical engineering. This reduction in workability may limit the practical application of higher modifier contents. Therefore, for future applications involving higher dosages (≥7.5%), it is recommended to introduce superplasticizers or adjust the water-to-cement ratio appropriately to compensate for the loss in fluidity while maintaining mechanical performance. Further optimization studies on mix design are warranted to balance workability and strength under such conditions. In addition, the reduction in flowability can be attributed to the following factors:

Compared to sand particles of an equivalent volume, red mud microspheres possess a higher specific surface area and compete with cement clinker for free water during the initial hydration stage. Moreover, the adsorbed water forms a hydration product layer that hinders further water diffusion into the microspheres, effectively reducing the actual water-to-cement ratio and decreasing particle lubrication. The introduction of microspheres alters the particle size distribution of fine aggregates, increasing the proportion of coarse particles and thus the inter-particle friction.

#### 3.1.2. Mechanical Properties of Modified Cement Mortar

**Figure 4:**
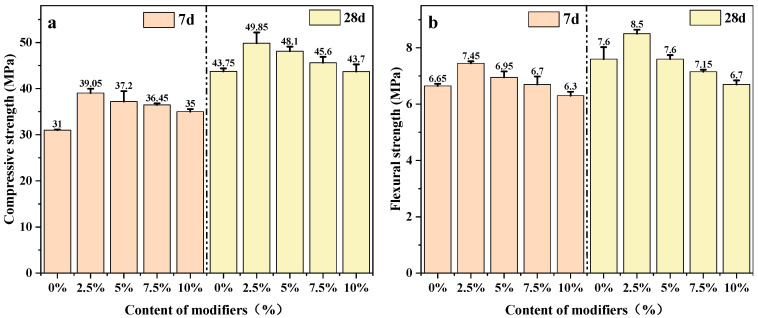
Mechanical properties of modified cement mortar graphs: (**a**) compressive strength; (**b**) flexural strength.

Figure 4 illustrates the flexural and compressive strength of all mortar samples at 7 and 28 days. The results show that at sand replacement rates ≤10%, the red mud microspheres yielded compressive strengths comparable to that of quartz sand, with the CMC-RM-SiO_2_-10% sample matching the baseline. However, as the replacement rate increased, the compressive strength gradually declined. This may be due to an excessive microspheres dosage, leading to increased porosity during hydration and hindering effective stress transfer. In addition, the high water absorption capacity of the microspheres reduced the amount of free water available for clinker hydration, thereby delaying or limiting the hydration process [25].

Among the tested compositions, CMC-RM-SiO_2_-2.5% exhibited the best mechanical performance, with its 28-day compressive strength being 3.8% higher than that of the control group. On the one hand, nano-silica grafting introduced additional nucleation sites and promoted pozzolanic reactions, generating supplementary C-S-H gel and densifying the interfacial transition zone. On the other hand, the hydrophobicity of the modified microspheres reduced water absorption and preserved more free water for cement hydration.

### 3.2. FTIR Analysis of Cement Mortar

**Figure 5:**
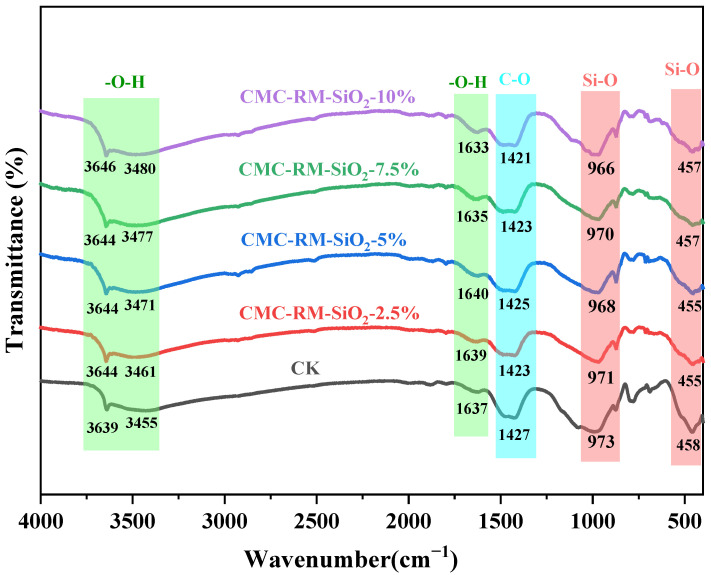
FT−IR plots of different modified cement mortars.

Figure 5 shows the FTIR spectra of different cement mortars curing for 28 days. The observed bands correspond to hydration products such as calcium carbonate (CaCO_3_), calcium hydroxide (CH), and calcium silicate hydrate (C–S–H). Absorption peaks near 3640 cm^−1^, 3450 cm^−1^, and 1640 cm^−1^ are attributed to the –OH groups in CH [26], indicating a significant CH content. The band at approximately 1420 cm^−1^ is due to the asymmetric stretching of the C–O bond in CaCO_3_, suggesting the presence of carbonate phases formed by the reaction of CH with atmospheric CO_2_ during hydration [27]. Peaks at 983 cm^−1^ and 458 cm^−1^ are related to the asymmetric stretching and bending vibrations of Si–O bonds in C–S–H [28], confirming the generation of considerable amounts of C–S–H gel. All spectra exhibit similar functional group types as the reference cement. This indicates that the inclusion of microspheres did not alter the types of hydration products or interfere with the chemical reaction mechanisms. Thus, the effects of microspheres are primarily physical, such as reducing free water and modifying particle gradation, rather than chemical.

### 3.3. XRD Analysis of Cement Mortar

**Figure 6:**
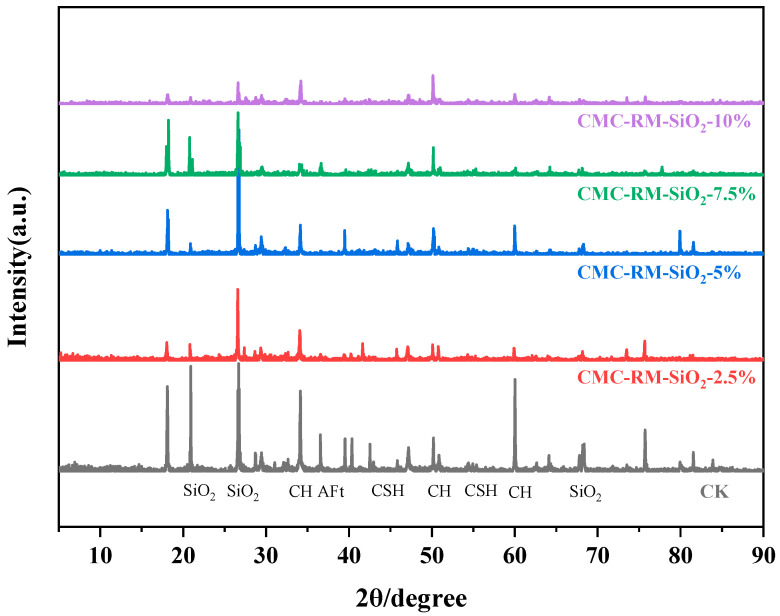
XRD patterns of different modified cement mortars.

Figure 6 displays the XRD spectra of different cement mortars after 28 days of curing. All samples exhibited a SiO_2_ diffraction peak at 2θ = 26.7°, along with characteristic peaks corresponding to CH, C–S–H, and AFt phases. No new crystalline phases were detected, indicating that the incorporation of red mud microspheres at different stages did not alter the chemical nature of the hydration products but only affected the intensity of the diffraction peaks [29]. In particular, for the CMC-RM-SiO_2_-2.5% sample, the CH peak near 2θ = 50° was significantly reduced, while the C–S–H-related peaks at 2θ = 42.5° and 54.9° were enhanced. This suggests that the modified microspheres promoted CH consumption and C–S–H generation, thereby accelerating the early hydration process [30]. The increased content of C–S–H gel not only improved the density of the interfacial transition zone (ITZ) but also reduced the formation of microcracks and defects, thereby enhancing the macroscopic mechanical performance of the modified cement mortar. This observation is consistent with the results obtained from the mechanical property tests in this study.

### 3.4. SEM Analysis of Cement Mortar

**Figure 7:**
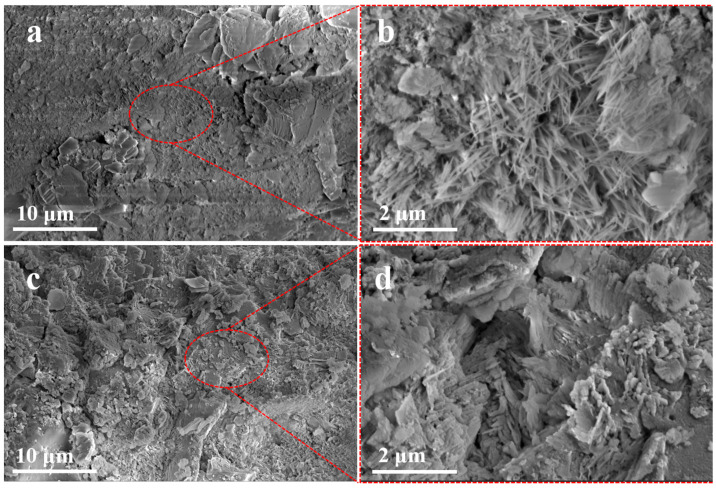
(**a**) SEM images of CK; (**b**) Partial magnification of CK surface; (**c**) CMC−RM−SiO_2_; (**d**) Partial magnification of CMC−RM−SiO_2_ surface.

Figure 7 shows the SEM images of different cement mortars after 28 days of hydration. In Figure 7a and c, the reference mortar surface exhibits abundant needle-like AFt crystals [31]. In contrast, the modified mortar shows a significantly reduced quantity of AFt, with a more uniform distribution of hydration products and a denser microstructure. The observed alteration suggests that introducing SiO_2_ could potentially optimize the pozzolanic interaction with CH, subsequently promoting the increased formation of C–S–H phases. This evolution contributes to microstructural densification, ultimately enhancing both compressive strength and chemical resistance in the cementitious composite.

### 3.5. TG Analysis of Cement Mortar

**Figure 8:**
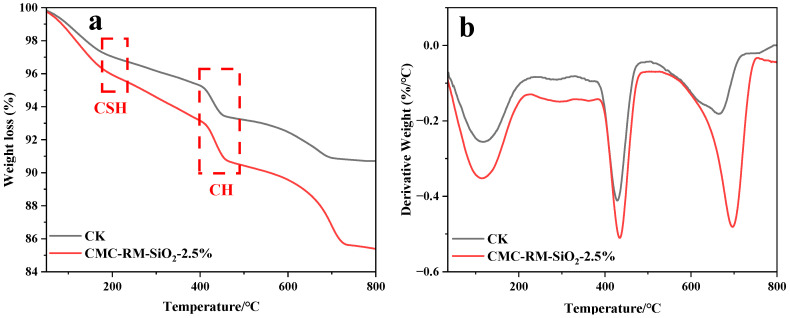
Weight loss curves of 28d cement mortar. (**a**) TG; (**b**) DTG.

Figure 8 and Table 3 present the thermogravimetric (TG) analysis results of the cement mortars after 28 days of hydration. Significant weight losses are observed at around 200 °C and 400 °C, corresponding to the thermal decomposition of C–S–H and CH, respectively [32]. The data in Table 3 show that the modified mortars exhibited greater mass loss than the reference mortar, indicating a higher content of hydration products. This suggests a more complete hydration process in the modified mortars, yielding more C–S–H and other phases that enhance strength and durability. In particular, the CMC-RM-SiO_2_-2.5% sample demonstrated greater weight loss than CK, which is attributed to the participation of SiO_2_ in the hydration reactions, consistent with the SEM observations.

### 3.6. Toxicity Leaching Analysis

The toxicity leaching concentrations of red mud, red mud microspheres, and cement mortar are presented in Table 4. Toxicity leaching tests were conducted in accordance with GB 5085.7-2019 [33] and HJ298-2019 [34]. The results showed that, except for the raw red mud, the concentrations of Cr, As, Pb, Co, Cd, and Na in the red mud microspheres and cement mortar were all below the limits specified in GB 8978-1996 [35], indicating their environmental safety. Notably, Cr and Pb were not detected in the two types of red mud microspheres, further confirming the encapsulating effectiveness of calcium alginate. By forming stable complexes, calcium alginate significantly reduces the mobility of heavy metals, thereby mitigating their potential environmental and cement-related risks.

### 3.7. BET Analysis of Cement Mortar

**Figure 9:**
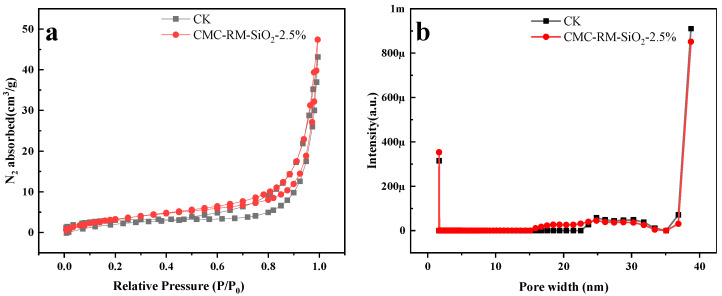
BET curves of different cement mortars: (**a**) N_2_ adsorption-desorption isotherms; (**b**) Pore size distribution diagrams.

Figure 9 shows the BET test results for various cement mortars after 28 days of curing. As shown in Figure 9a, all samples exhibit Type IV nitrogen adsorption–desorption isotherms, indicating a predominantly mesoporous structure [36]. According to Figure 9b and Table 5, the pore distribution of CMC-RM-SiO_2_-2.5% closely resembles that of the reference mortar. This implies that nano-silica grafting not only increases the specific surface area of red mud microspheres but also provides additional hydration sites. Such structural refinement contributes to the densification of the cement matrix and enhances its mechanical properties and overall stability.

## 4. Discussion

Among the existing treatment techniques for red mud, alkali-activation is a mainstream approach aimed at converting it into solidified materials, such as granules or powders. However, its industrial application is generally limited by its dependence on complex process parameters, thereby hindering the technology’s large-scale implementation. Tian et al. [37] reported that red mud contains relatively low amounts of reactive silica and alumina necessary for alkali activation. As a result, additional industrial wastes rich in reactive Si and Al are often required to enable sufficient activation, thereby increasing the complexity of the pelletization process. Moreover, Zhao et al. [18] found that curing temperature and curing time have distinct effects on the strength of the microspheres, indicating that alkali-activated pelletization lacks broad compatibility when applied to red mud from various sources and with different activator systems. In the context of aggregate preparation, the study by Sun et al. [38] revealed that conventional treatment methods typically involve high-temperature processing, leading to considerable energy consumption.

This study innovatively employed the ionic crosslinking reaction of sodium alginate in a saturated calcium chloride solution to develop a method for preparing red mud-based microsphere aggregates encapsulated with calcium alginate. Compared with conventional alkali-activated pelletizing techniques, this approach offers broader compatibility with red mud from different sources and batches. It eliminates the need for fine-tuning alkali activator formulations during the pelletizing process, thus providing greater practicality and wider applicability in engineering applications. This method consistently produces structurally intact microspheres, effectively overcoming the strict raw material homogeneity required by conventional methods. From an engineering perspective, this study provides a theoretical basis and technical pathway for exploring novel aggregate substitute materials.

In future research, we plan to explore more cost-effective alternatives. Additionally, the optimization of the dosage and process simplification will be considered to improve economic viability.

## 5. Conclusions

This study developed a novel red mud utilization strategy by preparing CMC-RM-SiO_2_ microspheres, in which red mud was encapsulated with calcium alginate and further modified by nano-silica grafting. The grafting effect of nano-silica was confirmed through microstructural characterization. The potential of using red mud microspheres as partial sand replacements in cement mortar was evaluated through performance tests and microstructural analyses. The key conclusions are as follows:

(1) Mechanical tests demonstrated that the inclusion of red mud microspheres significantly improved the compressive strength of cement mortar at both 7 and 28 days. The optimum mechanical performance was achieved at a 2.5% replacement level. Furthermore, CMC-RM-SiO_2_ mortars outperformed those incorporating unmodified red mud microspheres.
(2) XRD and TG analyses revealed that the nano-silica-modified microsphere surfaces provided additional nucleation sites for cement hydration, resulting in the increased formation of C–S–H gel and enhanced hydration kinetics.
(3) BET analysis showed that the addition of microspheres reduced the total pore volume of the mortar, leading to a denser microstructure that contributed to improved mechanical properties and enhanced structural stability.
(4) The mechanical performance results of modified cement mortars indicated that at replacement rates ≤10%, the contribution of red mud microspheres to compressive strength was comparable to that of quartz sand, with only a 3.1% reduction observed for CMC-RM-10% compared to the reference.

In general, the CAC-RM-SiO_2_ red mud microspheres developed in this study exhibit good feasibility as a partial replacement for standard sand, particularly at a low dosage level (2.5%), where they significantly enhanced the mechanical properties of cement mortar. However, to ensure the reliability and cost-effectiveness of their practical application, future research will focus on process optimization, long-term performance evaluation, and the validation of their effectiveness under real engineering conditions.

## Figures and Tables

**Table 1:** Table of chemical composition of red mud and silicate cement (wt %).

	CaO	SiO_2_	Al_2_O_3_	Fe_2_O_3_	MgO	SO_3_	K_2_O	Na_2_O	TiO_2_
Red Mud	15.00	14.80	18.50	27.50	0.16	2.14	0.35	13.5	7.03
Portland Cement	63.60	18.50	2.93	1.61	1.46	4.13	0.85	0.13	-

**Table 2:** Formulations of mixtures with different RM dosages added into the cement mortar.

Samples	Dosage (%)	Symbol	Cement (g)	H_2_O (mL)	Sand (g)	RM (g)
Reference Cement	-	CK	450	225	1350.00	-
CMC-RM-SiO_2_	2.5	RM-2.5	450	225	1316.25	33.75
	5	RM-5	450	225	1282.50	67.50
	7.5	RM-7.5	450	225	1248.75	101.25
	10	RM-10	450	225	1215.00	135.00

**Table 3:** Specific gravity of mass loss after 28 d of hydration of different cement mortars.

T/°C	Weight Loss (%)	
	CK	CMC-RM-SiO_2_-2.5%
100–200	2.973	4.061
400–500	2.179	2.925

**Table 4:** Toxicity leaching values of red mud, red mud microspheres, and modified cement mortar.

Sample	As	Cd	Co	Cr	Pb
RM	0.0964	0.0007	0.1807	0.1047	0.0019
CMC-RM-SiO_2_	0.0161	-	0.0093	-	-
CK	-	-	0.0169	0.0439	-
CMC-RM-SiO_2_-10%	0.0016	-	0.0323	0.0649	-
GB 8978-1996 [35]	0.0500	0.0050	1.0000	0.1000	0.1000

**Table 5:** BET results of different cement mortars curing for 28 d.

Sample	BET Surface Area (m^2^/g)	Pore Volume (cm^3^/g)
Reference cement	11.7026	0.001578
CMC-RM-SiO_2_-2.5%	14.0190	0.001374

## Data Availability

The original contributions presented in the study are included in the article, further inquiries can be directed to the corresponding authors.
